# Public Attitudes on the Ethics of Deceptively Planting False Memories to Motivate Healthy Behavior

**DOI:** 10.1002/acp.3274

**Published:** 2016-09-21

**Authors:** Robert A. Nash, Shari R. Berkowitz, Simon Roche

**Affiliations:** ^1^ School of Life and Health SciencesAston UniversityBirminghamUK; ^2^ College of Business Administration and Public PolicyCalifornia State University, Dominguez HillsCarsonCAUSA; ^3^ School of PsychologyUniversity of SurreyGuildfordUK

## Abstract

Researchers have proposed that planting false memories could have positive behavioral consequences. The idea of deceptively planting ‘beneficial’ false memories outside of the laboratory raises important ethical questions, but how might the general public appraise this moral dilemma? In two studies, participants from the USA and UK read about a fictional ‘false‐memory therapy’ that led people to adopt healthy behaviors. Participants then reported their attitudes toward the acceptability of this therapy, via scale‐rating (both studies) and open‐text (study 2) responses. The data revealed highly divergent responses to this contentious issue, ranging from abject horror to unqualified enthusiasm. Moreover, the responses shed light on conditions that participants believed would make the therapy less or more ethical. Whether or not deceptively planting memories outside the lab could ever be justifiable, these studies add valuable evidence to scientific and societal debates on neuroethics, whose relevance to memory science is increasingly acute. Copyright © 2016 The Authors *Applied Cognitive Psychology* Published by John Wiley & Sons Ltd.

Consider an important memory from your childhood, which inspires you to do great things or to be a better person. Now, imagine learning that this experience never truly happened and that in fact, the memory was artificially planted in your mind by somebody intent on pushing you to achieve your potential. How would you react: with outrage, perhaps, or with gratitude? In recent years, psychological scientists have recognized that certain false autobiographical memories could lead to improvements in people's lives and lifestyles (Bernstein and Loftus, 2009; Laney and Loftus, 2017). One implication is that deliberately planting such memories could in principle serve to benefit individuals and societies. But would the general public ever view such an intervention as acceptable? In this paper, we report the first systematic examination of how people weigh the ethics of using deception to plant ‘beneficial’ false memories, as a means to control people's unhealthy behaviors.

Most memory researchers conceive false memories as a normal by‐product of our flexible and reconstructive memory system, wherein recollections are altered each time they are retrieved (Newman and Lindsay, 2009; Schacter, 2001). Because the capacity for distortion is inherent in everyday memory processes, ‘persuading’ people to change their memories is far more plausible than most people typically believe (Nash, Wheeler, and Hope, 2015; Simons and Chabris, 2011). Indeed, in studies conducted over the past 20 years, researchers have used manipulation and suggestion to plant distinctive and emotional false childhood experiences into participants' memories—from taking a balloon ride, to being attacked by an animal, to committing a crime (Porter, Yuille, and Lehman, 1999; Shaw and Porter, 2015; Wade, Garry, Nash, and Harper, 2010). Together, these studies demonstrate that it is indeed possible to plant vivid and detailed memories of entire fictional experiences.

Memories play a crucial role in shaping our attitudes, decisions, and behavior (Biondolillo and Pillemer, 2015; Pezdek and Salim, 2011). Importantly, a wealth of evidence confirms that this role applies to false beliefs and memories, as well as to true beliefs and memories. For example, after developing false beliefs of having their ear uncomfortably ‘licked’ by a rogue Pluto character at Disneyland, participants in one study were subsequently less willing to pay for a Pluto souvenir (Berkowitz, Laney, Morris, Garry, and Loftus, 2008). In other contexts, false memories stand to have more severe consequences, such as mistaking a terminally ill family member's dying wishes (Sharman, Garry, Jacobsen, Loftus, and Ditto, 2008) or identifying an innocent suspect from a lineup (Garrett, 2011). In recent years, though, researchers have also explored the possibility that false memories can have positive behavioral consequences (Fernández, 2015; Howe, 2011).

The largest research program so far to explore the positive behavioral consequences of false beliefs and memories focuses on healthy eating (Bernstein and Loftus, 2009; Laney and Loftus, 2017). In these ‘food studies’, researchers have led adult participants to falsely believe in or remember either a negative childhood experience with food or drink (e.g. got sick after consuming it) or a positive experience (loved it the first time they tried it). These studies showed that false beliefs and memories influenced participants' current dietary preferences. For instance, people who developed false beliefs and memories of getting sick from consuming hard‐boiled eggs, dill pickles, strawberry ice cream, vodka or rum subsequently reported less interest in consuming those foods or drinks again (Bernstein, Laney, Morris, and Loftus, 2005a, 2005b; Clifasefi, Bernstein, Mantonakis, and Loftus, 2013). Similarly, people who developed false childhood beliefs or memories of loving asparagus reported a stronger preference for that food (Laney, Bowman Fowler, Nelson, Bernstein & Loftus, 2008; Laney, Morris, Bernstein, Wakefield, & Loftus, 2008). Perhaps even more compelling, participants who developed false childhood beliefs or memories of getting sick after eating spoiled peach yogurt actually ate less peach yogurt in an ostensibly unrelated taste test the following week (Scoboria, Mazzoni, and Jarry, 2008; Scoboria, Mazzoni, Jarry, and Bernstein, 2012). These latter studies demonstrate especially clearly that false beliefs and memories can influence behavior.

Suppose then that by using deception to plant false memories of certain childhood eating experiences, an expert could lead obese people to change their consumption of unhealthy foods. After the first of the food studies was published, the media were quick to speculate about this possibility, coining it the ‘False‐Memory Diet’ (Glassie, 2005). One could also envision many other hypothetical variants: Perhaps false memories could make people less scared of visiting the dentist (Pickrell et al., 2007) or make lazy people love to exercise. Researchers and practitioners within the field of psychotherapy have even discussed the viability of deliberately planting false memories as a way to resolve and treat psychological traumas. Indeed, since at least the late 19th century, there have been anecdotal—and arguably troubling—claims of therapeutic attempts and successes in this regard (e.g. Gravitz, 1994; Meyerson, 2010; Scheflin, 1997).

But would such deceptive and manipulative interventions ever be advocated in practice? And, more importantly, should they? Undoubtedly, the notion raises sizeable moral and ethical questions. There is of course long‐standing concern about the acceptability of certain psychological therapies that have the potential to evoke false memories (Loftus and Ketcham, 1994). This concern, however, typically relates to the idea that a therapist could intentionally or unintentionally plant false memories of childhood trauma. Few people, we hope, would seriously entertain the idea that it could ever be acceptable to plant false memories of child abuse. Perhaps some people would feel differently, though, about the acceptability of planting relatively benign, nontraumatic memories with the benevolent intention of improving someone's health and well‐being. These moral and ethical questions—irrespective of how legitimate memory‐planting therapies would ever be—are important to tackle as neuroethical and neurophilosophical perspectives assume increasingly crucial roles in the science of memory modification.

### The ethics of modifying memories

The ethical dimension of the false‐memory therapy scenario has not escaped researchers' or media commentators' attention. As Bernstein, Pernat, and Loftus (2011) point out, ‘A few false memories of loving asparagus or getting sick from strawberry ice cream may or may not outweigh the ethics involved in planting these memories’ (p. 1660). Whereas many publications contain similarly cautious statements about the ethics of planting memories outside of the lab (e.g. Clifasefi et al., 2013; Pezdek & Freyd, 2009), some take less equivocal positions. For instance, Sher (2000) suggested that ‘The possibility of use of implantation of ‘good' memories in psychotherapeutic practice should be explored…. [It] should be used under strict ethical and legal control to prevent possible abuses’ (pp. 628–629). Opposing this view, Childress (2015) cautioned ‘However useful the creation of false memories might be in engendering healthy behavior, it transgresses important ethical barriers and violates respect for persons, their dignity, and their autonomy. It would be morally perilous for a democratic society even to contemplate such manipulations as options’ (pp. 34–35). Notably, these latter positions demonstrate wildly contrasting perspectives.

Yet, more than a decade after the first of the food studies was published, there have been no formal attempts to explore people's attitudes toward the ethics of deceptive false‐memory therapy (hereafter, *FMT*). In contrast, there is considerable research on the ethics of artificially erasing or weakening bad memories. Drugs such as propranolol can ‘dampen’ the emotional content of traumatic memories without erasing them completely, and this pharmaceutical possibility has triggered rich academic debate surrounding the foreseeable misuse or overuse of so‐called “cosmetic neurology” (e.g. Henry, Fishman, and Youngner, 2007; Liao and Sandberg, 2008). Tenenbaum and Reese (2007), for instance, have argued that memory manipulation creates a conflict between the interests of society and of individuals. For example, they noted that people's memories of terrible experiences can be instrumental in establishing safeguards that prevent other people from suffering similar experiences in the future. Indeed, dampening memories of criminal acts could be considered tantamount to contaminating legal evidence, and legal scholars have debated whether people might therefore have a moral duty to remember traumatic events (Kolber, 2006). In one study, researchers examined people's attitudes toward memory‐dampening drugs and found that although most (54%) claimed they would want the choice of receiving such a drug if they were a victim of a violent crime, relatively few (18%) would actually take it (Newman, Berkowitz, Nelson, Garry, and Loftus, 2011). These findings might lead us to expect that people would feel no more easy about the notion of altering memories via FMT.

### Public attitudes on planting beneficial memories

So what are people's attitudes on the ethics of FMT? At present, there is no systematic evidence of public views, akin to those captured by Newman et al. (2011), about this issue. People's responses to moral dilemmas often involve a utilitarian calculation of the ‘greater good’, opting for whichever course of action seems to evoke least harm (Baron, 1998; Gleichgerrcht and Young, 2013). A utilitarian might believe, then, that any health intervention can be ethical if it improves well‐being, perhaps irrespective of how that improvement is achieved. People might furthermore argue that autobiographical memory is inherently reconstructive and that inaccurate memories are therefore entirely ordinary, adaptive, and unlikely to be harmful (Newman and Lindsay, 2009). In contrast, people might forecast that FMT would cause many kinds of harm. We know, for instance, that people's autobiographical memories are closely interrelated with their sense of self and personal identity (Wilson and Ross, 2003). People who consider this interrelationship might worry about the ethics of leading people to experience their own identities in ways that are inauthentic (Erler, 2011). Moreover, unlike memory‐dampening drugs, the effectiveness of FMT would likely hinge on deceiving the ‘patient’—often considered taboo and the epitome of unethical practice in healthcare (Childress, 2015; Miller, Wendler, and Swartzman, 2005). We might reasonably expect, then, that many people would judge FMT as equally taboo.

The current studies aimed to examine public perceptions about the ethics and acceptability of a hypothetical FMT, although we should stress that our aim was *not* to explicitly or implicitly endorse memory‐planting interventions. Participants from the USA and UK—two countries whose obesity rates are among the highest in the Western world (www.who.int)—read a hypothetical scenario involving the deceptive use of FMT to alter unhealthy behavior, and reported their perceptions of the acceptability of this therapy. A utilitarian view on the ethics of FMT should hinge on considerations about whether or not FMT would cause more harm than it prevents (Baron, 1998). Based on this reasoning, we predicted that people would report more favorable attitudes toward FMT if they were first reminded of the public health and socioeconomic burden of obesity. To test this prediction, half of the participants in study 1 read contextual information and statistics about the burden of obesity, whereas half did not. Furthermore, given that people tend to have a rosy view of their personal pasts (Newman and Lindsay, 2009) and would therefore presumably see negative memories as undesirable, we predicted that people would feel more favorably toward FMT if it involved planting positive rather than negative false memories. To test this prediction, half of participants read about a therapist who plants happy childhood memories, whereas the other half read about a therapist who plants unhappy childhood memories. Alongside examining these two contextual factors, in study 1 we also collected data on participants' desire to control the events that occur to them (Burger and Cooper, 1979). We predicted that FMT would seem particularly unpalatable to people with a stronger desire for control.

## Study 1

### Method

The protocols for both of the present studies were approved by the first author's school ethics committee.

#### Participants

A market research company recruited 922 adult residents of the USA or UK, who participated online in exchange for credits that they could use to purchase rewards (e.g. gift cards). Within each national sample, the company recruited participants using sex and age quotas to approximate national demographics. However, 164 participants failed our attention check (described shortly) and were excluded from analyses. The remaining 758 participants included 415 people who identified as females, 342 who identified as males, and 1 person who specified neither female nor male sex. The participants had an overall mean age of 48.7 (*SD* = 16.8, range = 18–92; see Table S1 in Supplementary Materials for further demographic details).

### Materials

#### Vignettes

Depending on participants' randomly assigned condition, they received one of four vignettes describing a therapist who deceptively planted false childhood memories to improve a person's healthy‐eating behavior (see Supplementary Materials for the full text). Specifically, we first manipulated whether participants received information about the socioeconomic consequences of obesity (context vs. no context given). Participants in the ‘context’ conditions read a paragraph about the public health and socioeconomic burden of obesity in the USA and UK. This paragraph included statistical estimates from peer‐reviewed sources of how obesity affects the incidence of disease, the financial costs of treating obese patients, and future forecasts of these statistics. Participants in the ‘no context’ conditions did not receive this paragraph.

In the text that followed, participants in all conditions were asked to imagine that they were morbidly obese and sought professional support from a therapist after failing to control their diet. To help the patient improve their healthy eating, the therapist recommended a new therapy that involved talking about childhood memories of food. After several sessions of this therapy, the participant saw that their diet improved and began to lose weight. Months after the therapy ended, though, the therapist got in touch to disclose that the therapy had used deception: They had used ‘false‐memory therapy’ to deliberately plant false memories with the intention of changing the participant's eating habits. The therapist, they learned, collaborated with the patient's family to verify that the suggested events never truly occurred. The memories that the therapist supposedly planted varied in valence (positive vs. negative) depending on participants' randomly assigned condition. Participants in the ‘positive’ conditions were told the therapist had planted false memories that as a child they loved trying new healthy foods, such as asparagus and broccoli. In contrast, participants in the ‘negative’ conditions were told the therapist had planted false memories of having been really sick as a child after eating unhealthy foods, such as ice cream and donuts.

#### Desirability of Control

Participants also completed Burger and Cooper's (1979) Desirability of Control Scale, a 20‐item measure of people's preference for having control over the events that happen to them. Participants rated themselves on items such as ‘I try to avoid situations where someone else tells me what to do’, using a scale from 1 (the statement does not apply to me at all) to 7 (the statement always applies to me).

#### Procedure

After giving informed consent and reporting their sex, age, and ethnicity, participants read one of the four randomly assigned vignettes at their own pace and without a time limit. Then, participants responded to several written statements. First, they considered how acceptable it would be for a therapist to use FMT to improve their own and other people's eating habits. Specifically, using scales from 1 (strongly disagree) to 7 (strongly agree), participants responded to the statements: ‘If I were obese, I think it would be acceptable for a therapist to deliberately plant false memories to improve my healthy eating habits and reduce my obesity’ and ‘I think it would be acceptable for a therapist to deliberately plant false memories to improve other obese people's healthy eating habits and reduce their obesity’. The order of these two statements was counterbalanced, and hereafter, we refer to them as the ‘Acceptable for Me’ and ‘Acceptable for Others’ questions, respectively. Next, participants were asked about the ethics and morality of FMT. 1Many academics treat ‘morality’ and ‘ethics’ as interchangeable terms, but others argue that there are important distinctions between the two. We asked our participants whether FMT would be both moral and ethical because we believed that some participants may see the term ‘ethical’ as aligning specifically with professional codes of practice and ‘moral’ as aligning with more absolute ideals of right and wrong. Two statements were presented with the stem ‘I think that deliberately planting false childhood memories to improve a person's healthy eating habits and reduce their obesity is…’, and participants made ratings first from 1 (completely immoral) to 7 (completely moral) and then from 1 (completely unethical) to 7 (completely ethical) [Correction added on 08 October 2016, after first online publication: In the preceding sentence, the Ethical question rating representations were wrongly swapped and have been corrected in this version.]. These are hereafter referred to as the ‘Moral’ and ‘Ethical’ questions, respectively.

On the next page of the survey, we gauged whether participants thought this therapy could actually work. They rated two statements, ‘I think that deliberately planting false childhood memories is…’ (1 = completely impossible; 7 = completely possible) and ‘Assuming that somebody did develop false childhood memories during this therapy, I think the chance of them changing their eating habits as a result is…’ (1 = very unlikely; 7 = very likely).

Next, we included an attention check question to ensure that participants had actually read the vignette. We asked, ‘In the scenario you read above, what kinds of foods did the therapist plant false childhood memories about?’ Participants saw four options in a random order: asparagus and broccoli (the correct answer in the positive conditions), ice cream and donuts (the correct answer in the negative conditions), eggs and pickles, or cookies and cheese. All participants who chose an incorrect answer were excluded from analyses.

Participants next completed the Desirability of Control Scale and were asked whether they had ever experienced concerns about their own weight and/or eating habits (Yes or No). Finally, participants received debriefing information.

### Results and discussion

We first wanted to learn whether participants thought it would be acceptable for a therapist to use a deceptive FMT on them if they were obese. Recall that all participants rated their agreement that FMT would be Acceptable for Me, from 1 (strongly disagree) to 7 (strongly agree). Participants provided an average rating of 3.93 (*SD* = 2.29, *Mdn* = 4), almost exactly at the scale midpoint. Likewise, when asked about the acceptability of using the therapy on other obese people (Acceptable for Others), the mean rating was 3.78 (*SD =* 2.23, *Mdn* = 4). Interestingly, the difference between these two means was significant: People felt that FMT would be more acceptable if used on themselves than if used on others, *t*(757) = 4.34, *p* < .001, *d =* 0.16, Wilcoxon *Z* = 4.61, *p* < .001. Participants were however somewhat less convinced that FMT would be moral and ethical: Their mean ratings were 3.48 (*SD* = 1.98, *Mdn* = 4) and 3.47 (*SD =* 2.04, *Mdn* = 4), respectively.

**Figure 1:**
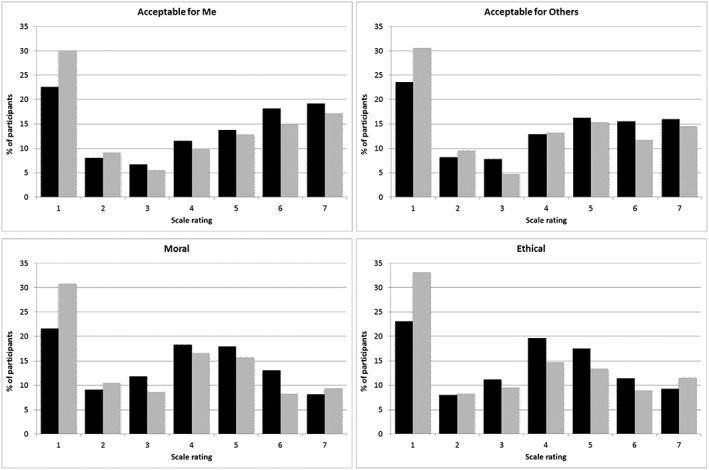
Distributions of ratings for the Acceptable for Me, Acceptable for Others, Moral, and Ethical questions, by participants in the positive memories condition (black bars) and negative memories condition (grey bars). For ‘Acceptable for me’ and ‘Acceptable for others’ questions, 1 = strongly disagree; 7 = strongly agree. For ‘Moral’ question, 1 = completely immoral; 7 = completely moral. For ‘Ethical’ question, 1 = completely unethical; 7 = completely ethical [Correction added on 08 October 2016, after first online publication: In Figure 1 caption, the rating representations for “Acceptable for me” and “Acceptable for others” were wrongly swapped and have been corrected in this version.].

Although participants' ratings of acceptability, morality, and ethicality averaged around the midpoint of the scales, their attitudes at the individual level were diverse. As Figure 1 illustrates, the modal response was that FMT would be entirely unacceptable, immoral and unethical, yet a sizeable minority of participants believed the exact opposite. Figure 1 also illustrates that people who were unfavorable toward FMT tended to have stronger views compared with those who were favorable. The following analyses attempt to identify some sources of systematic variance in these ratings.

#### Were people's attitudes influenced by the information they received?

We were primarily interested in the extent to which our experimental variables influenced participants' attitudes toward FMT. As illustrated in Table 1, a 2 (valence: positive vs. negative memories planted) × 2 (socioeconomic context: context vs. no context given) ANOVA on the Acceptable for Me ratings revealed that to a small but significant extent, planting positive memories was considered more acceptable than planting negative memories, *F*(1, 754) = 9.05, *p* < .01, *η* ^2^_p_ = .01, *d* = 0.22. The effect of adding socioeconomic context information was not statistically significant, *F*(1, 754) = 2.93, *p* = .09, *η* ^2^_p_ < .01, *d* = 0.12, nor was the interaction between valence and socioeconomic context, *F*(1, 754) = 0.28, *p* = .60, *η* ^2^_p_ < .001. The same pattern of results emerged for Acceptable for Others ratings, with a significant main effect of valence, *F*(1, 754) = 8.44, *p* < .01, *η* ^2^_p_ = .01, *d* = 0.21, no significant main effect of socioeconomic context, *F*(1, 754) = 1.16, *p* = .28, *η* ^2^_p_ < .01, *d* = 0.08, and no significant interaction, *F*(1, 754) = 0.29, *p* = .59, *η* ^2^_p_ < .001.

**Table 1:** Mean attitude ratings toward false‐memory therapy as a function of valence and socioeconomic context

	Condition			
	Negative memories		Positive memories	
	No context	Context	No context	Context
Acceptable for Me	3.58 (2.31)	3.77 (2.35)	3.99 (2.27)	4.36 (2.16)
Acceptable for Others	3.50 (2.26)	3.59 (2.30)	3.89 (2.21)	4.15 (2.11)
Moral	3.25 (2.03)	3.27 (2.04)	3.60 (1.98)	3.79 (1.83)
Ethical	3.27 (2.13)	3.26 (2.13)	3.60 (2.04)	3.77 (1.83)

Standard deviations in parentheses.

Looking to participants' ratings of the morality and ethics of FMT, our analyses revealed the same results as for acceptability. In both cases, there were main effects of valence [Moral *F*(1, 754) = 9.35, *p* < .01, *η* ^2^_p_ = .01, *d* = 0.22; Ethical *F*(1, 754) = 8.03, *p* < .01, *η* ^2^_p_ = .01, *d* = 0.21], but no main effects of socioeconomic context [Moral *F*(1, 754) = 0.56, *p* = .45, *η* ^2^_p_ < .001, *d* = 0.06; Ethical *F*(1, 754) = 0.24, *p* = .62, *η* ^2^_p_ < .001, *d* = 0.03] and no interactions [Moral *F*(1, 754) = 0.34, *p* = .56, *η* ^2^_p_ < .001; Ethical *F*(1, 754) = 0.38, *p* = .54, *η* ^2^_p_ < .001]. For all four attitude ratings (i.e. Acceptable for Me, Acceptable for Others, Moral, Ethical), nonparametric Mann–Whitney tests confirmed the significant main effect of valence (in all cases, *Z >* 2.88, *p* < .01) and nonsignificant effect of socioeconomic context (in all cases, *Z <* 1.70, *p* > .08). We also obtained the same pattern and significance of results, including the nonsignificant interaction effects, when we dichotomized each dependent variable into low (1–4) and high ratings (5–7) and conducted loglinear analyses. In short, these data suggest that attitudes toward FMT depend to a small extent on the type of memory that is planted but were not reliably changed when we emphasized the socioeconomic burden of obesity.

#### Who is most and least favorable to false memory therapy?

To explore whether participants' attitudes toward FMT differed systematically according to their demographic characteristics, we averaged Acceptable for Me, Acceptable for Others, Moral, and Ethical responses to form a composite ‘favorability’ scale (*α* = .97). In terms of nationality, UK participants were more favorable toward FMT (*M* = 3.94, *SD =* 1.98) than were those from the USA (*M* = 3.37, *SD =* 2.05), *t*(756) = 3.86, *p* < .001, *d* = 0.28, Mann–Whitney *Z* = 3.86, *p* < .001. Likewise, males were more favorable (*M =* 3.96, *SD =* 2.01) than females (*M* = 3.43, *SD =* 2.03), *t*(755) = 3.61, *p* < .001, *d* = 0.26, Mann–Whitney *Z* = 3.53, *p* < .001. There was also a weak, but statistically significant, negative correlation between age and favorability, such that younger participants were more favorable than older participants, *r*_spearman_ = −.09, *p* = .01. Attitudes toward FMT, it would seem, may differ quite systematically across different demographics, although it is important to note that all of these effects are small.

We also conducted two further exploratory analyses. One prediction we made was that participants who reported greater desirability of control would be less favorable to FMT. In fact, participants' favorability scores were not significantly related to their Desirability of Control scores, *r*_spearman_ = .07, *p* = .07. Moreover, favorability scores did not differ significantly between those participants who had experienced concerns about their weight and/or eating habits (*n* = 541; *M* = 3.60, *SD =* 2.06) and those who had not (*n* = 217; *M* = 3.82, *SD* = 1.98), *t*(756) = 1.35, *p* = .18, *d* = 0.11, Mann–Whitney *Z* = 1.23, *p* = .22.

#### Beliefs about the potential effectiveness of false memory therapy

Finally, we examined whether participants believed FMT could actually work. Overall, participants believed that it is somewhat possible to plant false childhood memories (*M =* 4.82, *SD* = 1.62, *Mdn* = 5) but were less sure whether these memories would change people's eating behaviors (*M =* 4.02, *SD* = 1.59, *Mdn* = 4). People's overall favorability ratings were positively correlated with their belief that it is possible to plant false childhood memories, *r*_spearman_ = .13, *p* < .001, and with their belief that false memories would change people's behavior, *r*_spearman_ = .43, *p* < .001. In other words, those people who were more inclined to believe that FMT could work were also more likely to find it acceptable, moral and ethical, although again, we emphasize that this association was weak.

## Study 2

The results of study 1 give insights into the variability of public attitudes toward deceptively planting beneficial false memories, but tell us little about people's reasoning. Why did some participants rate FMT as moral whereas others found it deeply immoral? To address this question, in study 2, we asked new participants to explain their attitudes toward FMT, and to consider any circumstances that might make FMT seem less or more acceptable, moral and ethical. We then coded the particular reasons that participants gave into different categories. Our categorization process was inductive and not guided by any *a priori* theory or coding scheme. However, in our description of the reasons given for and against FMT, we briefly relate each category to the four core principles outlined in the Universal Declaration of Ethical Principles for Psychologists (UDEPP), which underpin professional psychologists' ethical decision‐making. These are as follows:

1. *Respect* for the dignity of persons and peoples. This principle emphasizes the inherent worth of individuals irrespective of their social, racial, or individual identities and characteristics. It also includes considerations of people's right to provide free and informed consent, to privacy, and to fair and equal treatment.
2. *Competent caring* for the well‐being of persons and peoples. This principle emphasizes the importance of providing benefits to people and of either doing no harm or minimizing harm—often referred to as the ideals of beneficence and nonmaleficence, respectively.
3. *Integrity*, which emphasizes the fundamental goal of communicating honestly and openly, and also the importance of identifying and mitigating potential biases, prejudices and conflicts of interest.
4. *Professional and scientific responsibilities to society*, which involves considering how to improve scientific knowledge and understanding, and how to use this understanding responsibly and ethically to broadly serve the well‐being of society (International Union of Psychological Science, 2008).

### Method

#### Participants

A market research company recruited 240 participants who took part online in exchange for credits that they could use to purchase rewards. Exactly half were residents of the USA, and half were residents of the UK. Recruitment took place according to quotas to approximate national demographics for age and sex (see Table S2 in Supplementary Materials for demographic details of the sample). Our overall sample included 124 people who identified as females and 116 who identified as males. The participants had a mean age of 46.5 (*SD* = 17.2, range = 18–84).

#### Materials and procedure

All participants consented to take part and provided their demographic details before reading one of two vignettes. Both vignettes began with a general description of the rationale for the hypothetical FMT. Participants were asked to imagine that in the future, FMT exists and can be used to improve various health behaviors. Next, the vignettes contained the identical obesity scenarios as in the no‐context conditions of study 1, and we again experimentally manipulated whether the therapist planted positive or negative false memories in the fictional patient.

After reading one of the two randomly assigned vignettes, all participants typed open‐text responses to four questions. First, they were asked whether it would be acceptable, moral and ethical to deliberately plant false childhood memories to improve a person's health‐related behavior. They were then asked to describe any factors they might take into account when making this judgment, whereby they might think ‘It would be unethical, unless…,’ or ‘It sounds acceptable, but not if….’ On the next page, the other two questions pressed participants for their thoughts on whether two specific circumstances would make FMT less or more acceptable, moral and ethical: (1) if the therapist never disclosed the deception to the patient after their therapy ended and (b) if somebody other than a therapist—such as a parent or a teacher—planted false memories to change a person's health‐related behavior. After typing responses to these four questions, participants answered the ‘Moral’ and ‘Ethical’ scale‐rating questions from study 1, but with the stems re‐worded to ‘I think deliberately planting false childhood memories to make people behave in more healthy ways is…’ (1 = completely immoral/unethical; 7 = completely moral/ethical).

#### Data coding

Two researchers (R1 and R2) independently examined the responses to the first two open‐text questions, blind to participants' experimental condition and demographic details. Through discussion, R1 and R2 then used a content analytic approach to identify and iteratively refine broad ‘themes’ of reasons why the therapy was considered unacceptable or acceptable (listed shortly and in Tables 2 and 3). Next, R1 coded each participant's responses into as many of these themes as appropriate. In parallel, R1 and R2 used the same process to generate a second list of themes, signifying circumstances that participants said would affect the ethics of FMT (listed in Table 4). Again, R1 coded each participant's responses into as many of these themes as appropriate. For each of the latter two open‐text questions—about the ethics of failing to debrief the patient and of the use of FMT by nonprofessionals—R1 simply coded each response into one of three *a priori* categories: either (1) this scenario is generally less acceptable; (2) this scenario is generally more acceptable; or (3) this scenario is no different in acceptability, or the response was ambivalent or ambiguous. To assess inter‐rater reliability, a research assistant independently coded 20% of responses, blind to R1's judgments. We report reliability statistics in the respective sub‐sections of the text. Because reliability was good across all categories, all analyses are based on R1's coding.

**Table 2:** Reasons why participants believed that false memory therapy would be unacceptable

Reason category	Proportion of responses	Illustrative quotes
Consequences	37%	“*I think planting false memories is a dangerous practice. Perhaps the false memories of the patient in the previous example would give them some new sort of trauma.*” “*Memories that we can recall of real events that happened to us are what makes us individuals, if you take that away and implant false memories that person will lose sight of who they really are.*” “*I think I would feel manipulated and would lose my trust in the therapist. It is a situation where the client may feel they are baring their soul to a therapist and as such I would want to feel complete trust in the therapist.*”
The ends do not justify the means	32%	“*This method is not different than lying to someone and [then] excusing it [by] explaining why you lied. This does not undo the breach of trust that can go much further than the issue immediately at hand.*” “*I feel rather uneasy at the idea of people accessing my mind and tinkering with my memory.*”
Potential for abuse	14%	“*Far too dangerous. The first application I can see would be to persuade gay people they "ought" to be heterosexual. How long before the ruling party used it to "cure" people who voted for the opposition?*” “*This kind of 'treatment' could be very dangerous e.g. the person [might] have memories implanted suggesting that he enjoys eating cabbage every day instead of fast food, followed by a memory telling him that he had already decided that once he reached a certain weight he was going to Syria to join the IS or is going to stand in Trafalgar Square and cut his own throat.*”
Lack of consent	10%	“*This method distorts the concept of informed consent by putting the cart before the horse. Being informed after the fact doesn't ethically serve the purpose of protecting people's interests.*”
Practical doubts	8%	“*It's not likely childhood memories would stop someone from eating something if they have plenty of adult memories which tell them the opposite anyway.*” “*Surely there would be a very high risk of once the deception is exposed that the person may well regress in terms of their eating behaviour.*”
Better alternatives	7%	“*A better way to address this would be to try to get to the psychological root causes and reframe the way the person sees food, and change their habits via this method/approach instead.*”
Free will	3%	“*People have the choice on whether they eat a healthy diet or unhealthy food that make them obese, by changing a person's memories you are actually taking that choice away from them as they will act on memories and feelings. We do not have the right to change people in any way shape or form, we cannot act as gods and make those sorts of decisions.*”

**Table 3:** Reasons why participants believed that false‐memory therapy would be acceptable

Reason category	Proportion of responses	Illustrative quotes
The ends justify the means	36%	“*Yes I think this would be great. If it helps with your health surely that's the most important thing. [Too] many people worry about things that aren't as important as your health.*” “*If we are going to talk about morals then in my opinion someone who is abusing their body by being obese due to overeating or having alcoholism due to an addiction then it shouldn't really matter if a small trick has been used to help them improve themselves.*”
Increasing treatment options	6%	“*I think, generally, people should be allowed to do what they want with [themselves] provided they don't harm others and that we should seek to maximize their ability to make choices, with few restrictions, even if those choices might be an illusion.*”
Some people need support	6%	“*Many obese people will tell you that they need as much help as they can get as the reason they are in that position is because they have gotten out of control. Like drug abuse we don't expect these people to help themselves most of the time we put them in rehab. These people need something more powerful [than] their addiction something that triggers their [subconscious] to curb their cravings. Therefore I would believe this procedure to be deemed as acceptable.*” “*Sure it would be ethical, I know someone very close to me that is up to 540 lbs and will soon be a diabetic, nothing is getting through to her, she will shorten her life, if this type of therapy would work, I would be all for it, it would save her life. We love her and want what's best for her.*”
No harm would be done	5%	“*I don't think it is wrong at all and very acceptable. What harm can this do?*” “*I think that it is acceptable to plant false memories. Memories are very subjective, and people remember things differently than how they actually happened already.*”
No worse than alternatives	5%	“*Many medical treatments involve taking drugs or having surgical operations. These involve putting real things into the body. Sometimes they do not turn out beneficial and may even result in more harm than good. So, just putting false thoughts into someone's thoughts does not seem nearly as invasive or potentially harmful.*”

**Table 4:** Circumstances that participants believed would influence the acceptability of false‐memory therapy.

Reason category	Proportion of responses	Illustrative quotes
Gaining informed consent	23%	“*If the person is OK with having false memories implanted, then it's fine. But they should get the choice to do so, and not have it done without their knowledge*” “*To make it acceptable: informed consent of the patient, or a contract that covers more than one possible practice so false memories are a possibility but not a foregone conclusion (this is probably impossible, which is partly why I don't think it could/should be done)*”
Professional oversight	16%	“*Subject to a proper ethical and moral code of practice being put in place I think this would be a perfectly acceptable and excellent way of tackling obesity and other [health] problems and phobias.*” “*Psychologists and/or therapists should have to go through rigorous testing before being taught this technique and allowed to use it.*”
Patient factors	14%	“*I believe false memory therapy is only unethical if the patient is not suffering from a life threatening condition […] if it was used because someone was slightly overweight for example and just wanted to look better i.e. no direct threat to their health, then it would be unethical. It's relative to the situation of the patient.*”
Contextual factors	12%	“*There are lots of things that go wrong in a person's life, some things that make it hard to go on. Like my sister [passed] away, as unbearable as that was for me and my family, especially my mom. I don't think it'd be right to rewrite something like that in someone's life. As painful as it is, she is my sister, I wouldn't want my memories changed of her.*”
Evidence	9%	“*It's acceptable [… if] it is found that actually long‐term false memories are very very unlikely to cause any harm.*” “*If you can still do things you normally do daily without any mental or physical side effects, then I see no factors that would make this unacceptable.*”
Debriefing	3%	“*I don't believe that it is immoral or unethical in any way provided the person being treated understands at the end that they are going to get long term benefit from the treatment.*” “*I believe as long as the person is informed that [it] was false afterwards and is shown what good it has done then [there] is no harm done.*”

### Results and discussion

Our analysis revealed seven key reasons why people tended to find FMT unacceptable, and five key reasons why people tended to find FMT acceptable. Tables 2 and 3 provide illustrative quotes for each of these key arguments against and for FMT, respectively, and some additional quotes are included in the main text. In total, 93% of participants' responses fit at least one category; the remainder were too unclear or nonspecific to classify. Inter‐rater agreement ranged from 81 to 98% across these 12 categories (Gwet's AC_1_ from .64 to .98).

### Reasons why FMT is unacceptable

#### Consequences

As Table 2 shows, the most common overall arguments against FMT, seen in 37% of responses, involved concerns about the consequences or dangers of planting false memories. In other words, the goal of nonmaleficence (i.e. minimizing or avoiding harm) was a fundamental guide for these people's reasoning, as reflected in the UDEPP core principle of ‘competent caring’. Many participants feared unspecified side effects that might plausibly arise from FMT, but others had more specific concerns, as follows and as illustrated in Table 2.

Psychological consequences. Some people argued that planting false memories could harm the patient's psychological well‐being, either because they believed merely having a false memory could in itself cause psychological harm, or because they believed there may be psychological effects of discovering that a childhood memory was false:

People may not cope with the end result if they have so many good memories to then find out [they're] not true, we are a nation of stress eaters so [therefore] it may turn people very depressed.

Authenticity. Some participants shared a concern about the effect that false memories would have on the authenticity of patients' lives. Several pointed out that memories are intricately tied up with personality and identity in this sense, and argued that being grounded in reality is a critical component of any successful therapy.

Social consequences. Other participants feared social repercussions of planting false memories, particularly how those memories—or discovering the deception—might affect the patient's relationships with the therapist or with family members:

The individual might begin to blame their parents for allowing them to overeat as a child, and causing the adulthood obesity.

#### The ends do not justify the means

The second largest principal argument against FMT (32%) was that the integrity of therapeutic methods is more important than any potential benefits. In the context of professional ethical principles, these people implied that even if FMT could meet the goal of beneficence (i.e. providing benefits), as mirrored in UDEPP's principle of ‘competent caring’, achieving this goal would nevertheless be outweighed by serious concerns over the principle of ‘integrity’. Many participants made general comments to this effect, some of whom specifically pointed to the perceived immorality of lying to people in healthcare contexts or indeed in general:

It would not be acceptable because it would be lying to the person. Tell them the truth. I don't really know what else my thoughts are except that it would be lying, and lying is wrong.

Participants in this category also frequently alluded to a general sense of distaste with ‘tampering’ with people's minds:

I think it would be very unethical and immoral to do anything close to this. Our minds should not be modified in this way.

#### Potential for abuse

The next most common reason given against FMT—expressed by 14% of participants—was a concern that it could ultimately be used for purposes more nefarious than improving public health. These responses emphasize again the core ethical principle of ‘integrity’, under which the UDEPP specifies ‘not exploiting persons or peoples for personal, professional, or financial gain’ (International Union of Psychological Science, 2008). But they are also well captured by the principle of ‘Professional and Scientific Responsibilities to Society’, under which UDEPP includes ‘the discipline's responsibility to use psychological knowledge for beneficial purposes and to protect such knowledge from being misused’ (International Union of Psychological Science, 2008). Our participants envisaged several examples of possible nefarious uses of FMT, for example:

If you could convince me I ate certain things and got sick you could convince me I killed someone.

#### Lack of consent

In the ethical domains of ‘respect’ and ‘integrity’, some participants unsurprisingly mentioned the lack of informed consent as a reason why FMT is unacceptable (10%). It may seem more surprising that as few as 10% of people were classified into this category; however, note that many other people suggested that gaining consent could make the therapy more ethical. These kind of ‘less or more ethical’ responses were coded separately in the ‘circumstances’ analysis, described shortly.

#### Practical doubts

Some participants expressed doubts about whether FMT could actually work (8%), which of course would present a substantial concern in the ethical domain of ‘competent caring’. In very few cases, these doubts regarded the actual plausibility of planting false childhood memories, but in more cases, they concerned the likelihood that false memories would lead people to change their behavior or about the longevity of this behavior change after the deception were revealed.

#### Better alternatives

A small minority of people (7%) felt that FMT would be unacceptable partly because they believed that other less‐questionable methods would be equally or more effective for improving healthy behavior. These concerns would once again align with the UDEPP principle of ‘competent caring’.

#### Free will

Finally, 3% of participants had concerns about the extent to which planting false memories would rob patients of the free will to control their own behavior. These participants' concerns seem to relate to the considerations of human dignity encompassed within the core ethical principle of ‘respect’. But they also once again relate to ‘competent caring’, insofar as that principle concerns ‘respect for the ability of individuals, families, groups, and communities to make decisions for themselves and to care for themselves and each other’ (International Union of Psychological Science, 2008).

### Reasons why FMT is acceptable

#### The ends justify the means

Among those participants who expressed at least partly favorable attitudes toward FMT, the majority suggested that the ends *do* justify the means (36% of the total sample). That is to say, like many of the people described in the preceding sections, these people recognized a tension between the principles of ‘integrity’ and the beneficence aspects of ‘competent caring’. However, unlike those described in the preceding sections, these people saw beneficence as being the primary index of ethical practice, rather than integrity. Some in this category argued simply that helping people and potentially saving lives should always be the foremost concern, whereas others explicitly considered the potential risks but determined that these were outweighed by the potential benefits.

#### Increasing treatment options

Focusing on another aspect of ‘competent caring’, participants sometimes argued (6%) that FMT would increase the diversity of healthcare options available and that it should be up to individuals or their families to determine which options to pursue.

#### Some people need support

Similarly, some participants (6%) argued that people are not always able to control their own behavior and therefore often need external support to do so. These concerns once again resonate with the principle of ‘competent caring’, but may also be interpreted in terms of the principle of ‘respect’ insofar as it concerns ‘fairness and justice in the treatment of persons and peoples’ (International Union of Psychological Science, 2008). Interestingly, among the participants in this category were some who explicitly claimed that they would wish to receive FMT, either for someone they knew or for themselves:

As someone who has struggled with her weight all her life, I wouldn't be upset at all to have someone help me in this manner. I would think that some people would resent it; although I can't imagine why, if the therapy worked.

#### No harm would be done

Considerations about nonmaleficence (under ‘competent caring’) were relevant to some of the arguments in favor of FMT, not just those against. Specifically, a minority of participants (5%) felt that planting false memories could simply do no harm, or that because memory is naturally reconstructive anyway, having false memories is normal.

#### No worse than alternatives

Finally, 5% of participants argued that FMT would be similar to, no worse than, or even better than alternative interventions that already exist. Relative appraisals of nonmaleficence and beneficence, encompassed in ‘competent caring’, were therefore the apparent drivers behind these participants' reasoning.

In sum, diverse arguments underpinned participants' beliefs for and against using FMT. Whereas many of these arguments highlighted differences of opinion over whether or not the ends can truly justify the means, others shed light on people's beliefs about memory and their valuing of authentic recollection. We found little evidence that our valence manipulation (positive vs. negative memories) influenced the types of arguments people gave. In fact, comparing how frequently the seven ‘against’ and the five ‘for’ reason categories were used across conditions, there was just one significant difference even without correcting for multiple comparisons. Specifically, participants in the negative condition seemed somewhat more likely to use a ‘better alternatives’ line of argument against FMT (10%), compared with those in the positive condition (4%), χ^2^ = 4.35, *p* = .04, φ = .14. For all other comparisons, χ^2^ < 2.55, *p* > .11, φ < .11.

### Circumstances that would influence the acceptability of FMT

Whether or not people found the idea of FMT appealing, their views might be contingent on factors not accounted for in the vignettes. Our data revealed six types of consideration that people said would affect the ethics of FMT; these are summarized in Table 4 alongside illustrative quotes. In total, 54% of participants described at least one of these considerations; Inter‐rater agreement ranged from 75 to 96% across the categories (Gwet's AC_1_ from .65 to .93).

#### Gaining informed consent

The most commonly mentioned circumstance was gaining consent: 23% of participants felt that the therapy could be (more) acceptable if it was possible to gain informed consent from patients or their families. Some of these, of course, were the same people who also specified the lack of consent as a reason why FMT was unethical in the form it was described. Interestingly, several participants suggested ways of gaining partial consent without completely disclosing the methods, which they believed could make the therapy more acceptable:

It's acceptable [… if] a person consents to ‘deceptive’ or ‘risky’ treatments, even if they don't know precisely what they are.

#### Professional oversight

For some participants (16%), the acceptability of FMT would depend on the extent and quality of the professional standards in place for monitoring and regulating this therapy, and on the therapist's motives.

#### Patient factors

A subset of participants (14%) felt that the therapy would be more acceptable for some patients than others, especially insofar as the severity of their health problem was concerned. Among these participants, some said that FMT would only be acceptable if all other options had been exhausted:

Other treatments should be attempted first, obviously, but if all of them fail, and there is a real danger for [irreversible] damage, then this method of treatment should be attempted. I suppose I would look at it as a sort of last resort tactic.

#### Contextual factors

Although our valence manipulation did not systematically alter participants' arguments, participants did occasionally indicate that the acceptability of FMT would depend on what kinds of memories were implanted and for what purpose (12%):

Don't soil a third party's reputation or implant memories that could be potentially traumatic, such as false memories of abuse from a friend or family member.

It would not be acceptable under any normal circumstances unless requested by the subject although as a way of [rehabilitating] a criminal […] it may be considered.

#### Evidence

A small group of people (9%) said that they would take into account what was known about the effectiveness and side effects of the therapy.

#### Debriefing

Finally, only 3% of participants spontaneously said that the acceptability of the therapy would depend on the nature of the debriefing.

Together, these six circumstances tell us that although participants often had strong views on the idea of FMT, not all of these views were unequivocal, and in fact, a majority felt that planting false memories outside of the lab could be less or more acceptable under certain conditions.

### Would it be less or more acceptable if…?

Next, we examined whether participants felt it would make a difference if the patient were never debriefed, or if false memories were planted by nonprofessionals such as a parent or teacher. The analysis showed that 23% believed failing to debrief the patient would make the therapy less acceptable, whereas 7% felt that it would make the therapy more acceptable (inter‐rater agreement = 88%, Gwet's AC_1_ = .83; see Table 5 for illustrative quotes). In total, 28% said that it would be less acceptable for nonprofessionals to plant false memories, whereas 4% said that this could be more acceptable than the therapeutic scenario (inter‐rater agreement = 83%, Gwet's AC_1_ = .76).

**Table 5:** Illustrative quotes from participants regarding alternative forms of the ‘false‐memory therapy’ scenario

	Makes it less acceptable	Makes it more acceptable
Failing to debrief the patient	“*It would make it less ethical. Because you would in essence be, to a degree, rewriting that person's very history. Their life. The original method is still morally ambiguous, however at least it attempts to undo some of the potential harm with honesty a bit later on. This method does not have that feature.*” “*This makes it more unethical. Your memories would need to be a lie for the rest of your life. Memories are far too important to our self‐identity.*”	“*I think if a therapist has made false memories for a patient then they should not be taken away once the patient is better, this would be unethical and not fair on the person who has been given false memories in the first place. This could undo all the work done in the first place.*” “*I think that it would be far better and much more kinder to the patient to allow them to continue believing everything they had previously been told.*”
Planting of ‘beneficial’ false memories by nonprofessionals	“*Less acceptable because it's being presented as a kind of medical treatment and these people wouldn't have medical degrees, held to oaths, and wouldn't have those degrees or accreditation taken away if they arbitrarily practiced it. Additionally, parents and teachers are definitely going to abuse it, there's just no question.*” “*No I think this should only be done by a professional. Teachers and parents aren't skilled enough for mind control.*”	“*If a parent were to plant these memories you wouldn't necessarily look at them as unethical as you are trying to improve your [child's] health. Parents frequently tell little white lies to children to get them to finish food or finish homework. You are not placing your trust in a professional by having your parents do this.*” “*I feel it would be more acceptable if it were a parent as I would have absolute trust in a parent's actions being completely for my benefit with only my good as their justification.*”

### Scale‐rating data

Finally, we analyzed participants' scale ratings, averaging their ratings of the morality and ethicality of FMT to form a favorability index (*α* = .97). Looking at these scores, there was no significant main effect of valence (positive *M =* 3.52, *SD =* 2.04; negative *M* = 3.20, *SD* = 1.95), *t*(238) = 1.26, *p* = .21, *d* = 0.16, Mann–Whitney *Z* = 1.25, *p* = .21. However, our UK sample was again significantly more favorable toward FMT (*M =* 3.67, *SD =* 1.84) than was the US sample (*M =* 3.09, *SD =* 2.12), albeit this was once again a small effect, *t*(238) = 2.28, *p* = .02, *d* = 0.29, Mann–Whitney *Z* = 2.54, *p* = .01. The main effect of participant sex was not significant (females, *M =* 3.29, *SD* = 1.98; males, *M* = 3.47, *SD* = 2.03), *t*(238) = 0.71, *p* = .48, *d* = 0.09, Mann–Whitney *Z* = 0.68, *p* = .50, and nor was the correlation between favorability and participant age, *r*_spearman_ = −.10, *p* = .14.

## General Discussion

Using deception to plant ‘beneficial’ false memories outside of the laboratory could in principle have many conceivable applications. Indeed, there is good reason to believe that forms of this intervention have been used in certain niche (and perhaps troubling) psychotherapies for many decades (Gravitz, 1994; Scheflin, 1997). Yet, the idea raises serious ethical dilemmas. As the evidence mounts showing that false memories can promote positive behavior change, the present studies are the first to explore public attitudes on this contentious issue.

Both studies highlight a wide range of reactions to the notion of a hypothetical false‐memory therapy. In the majority were people who found the idea of FMT horrific, sinister and an affront to individuals' freedom to control their own lives. These participants described quite diverse lines of reasoning, drawing upon the UDEPP ethical principles of respect for people's dignity, of competent caring for people's well‐being, and of integrity. These people often foresaw diverse unwanted side‐effects of planting memories, and imagined that sanctioning false memory treatments would begin a slippery slope toward serious abuses of power and of people. In contrast, a substantial minority of participants were highly enthused about this fictional therapy, and even believed that it would be more attractive than existing health or medical interventions. These participants tended to base their reasoning mostly on considerations about competent caring for people's well‐being, specifically in terms of providing beneficence. That is to say, they often took a utilitarian approach and felt that the potential harm presented by this intervention would be minimal compared with the benefits it might deliver. It is interesting to note the relative absence of arguments, for or against FMT, which emphasized the fourth UDEPP principle, namely professional and scientific responsibilities to society. Our participants tended to focus their ethical reasoning at the level of the individuals who might hypothetically receive FMT, rather than at the societal level; this result may shed some light on why our context manipulation in study 1 had no discernible effects on participants' judgments.

Among those participants who felt strongly against the idea of FMT, most had important practical concerns, citing, for example, individuals' inability to give consent, and the inappropriateness of using deception in healthcare contexts. These concerns were likely fundamental in setting our participants' consensus generally against the acceptability of FMT. Much laboratory research on false memories involves deceiving participants to some extent, and it seems reasonable to assume that the use of deception would likewise be a crucial component of any viable FMT. However, the accuracy of this assumption is an open question, especially when we consider the results from randomized medical trials in which patients saw health improvements despite being truthfully informed that they were only receiving an inert placebo (see Kaptchuk et al., 2010, for a striking example). It would therefore be interesting to know the extent to which the consensus on FMT might shift if the hypothetical patient gave full informed consent and no deception were employed.

It is unlikely, though, that all of people's concerns about the ethics of FMT would be resolved by avoiding deception. Many of our participants' additional concerns—particularly those regarding the possibility that false memories themselves could have negative consequences—offer broader insight into people's understanding of memory distortion. In particular, these responses illustrate the value that people place upon authentic recollection, that is, remembering in ways that are ‘true to ourselves’ and that afford control over our own identities (Erler, 2011). In this respect, our findings are compatible with those of Newman et al. (2011), in showing that people typically resist the notion of tampering with memories, even when doing so could serve to benefit the individual. However, like in Newman et al.'s study, the present data also suggest that people do not always place memory authenticity above all other considerations. Indeed, although the majority consensus in both present studies fell against the use of FMT, both datasets also highlight circumstances under which participants felt planting memories could be (more) acceptable. For instance, in study 1, people were rather more comfortable with planting positive memories compared with negative memories. This small effect was consistent but not significant in study 2. That study did reveal, though, that many people's attitudes toward FMT depended on considerations such as the type of memory planted, the severity of the patient's health issues, and the safeguards in place to protect patients from harm.

It is worth emphasizing that the participants in these studies considered a hypothetical scenario for which they likely had no preconceived beliefs or attitudes. Naturally then, the difficulty of affective forecasting is relevant here (Wilson and Gilbert, 2003), and it is impossible to know whether people's stated beliefs would be similar if FMT were a real and viable treatment option for a friend or relative. Nevertheless, these data serve as a snapshot of people's instincts on the acceptability of deceptive memory manipulation. Views on the ethics of memory enhancement typically evolve over time; indeed, even the use of mnemonics and written memory aids has been considered immoral within some societies at particular points in history (Madan, 2014). As public awareness of memory's reconstructive properties increases, it is interesting to consider whether views toward FMT might similarly change over time. In study 1, the people who found the idea of FMT most acceptable tended to be those who believed strongly (in line with the scientific literature) that planting false childhood memories is possible. This finding might lead us to speculate not only that attitudes on neuroethical dilemmas such as these could be more liberal among cognitive scientists than among the general public, but also that public attitudes could become more liberal as public understanding of memory functioning grows. This prediction is bolstered by our finding that younger participants—who typically have more accurate beliefs about memory than do older generations (e.g. Simons and Chabris, 2011)—were somewhat more in favor of FMT. Whether a shift in attitudes over time would be desirable is, of course, a matter of opinion, yet the possibility further underscores the importance and timeliness of mapping this contentious ethical terrain.

The present studies supplement the literature on neuroethics and, we hope, will stimulate broader ethical, legal, psychological and philosophical discussions concerning the implications of beneficial false memories. Despite encountering diverse viewpoints in these studies, on the whole, there does not appear to be a strong appetite for health‐boosting false‐memory therapies. Even if, in principle, planting false memories could reduce people's yearning for fatty or sugary foods, doing so would nevertheless leave a sour taste in the mouths of many.

## Supporting information

Table S1. Demographic characteristics of the study 1 participants. Figures outside of parentheses represent raw numbers of participants.
Vignette used in study 1 (text in italics was only presented to those participants in the ‘context’ conditions. Text highlighted in bold indicates those elements that differed between the ‘positive’ and ‘negative’ conditions. In the actual study materials, all text was presented in regular typeface).
Primary questions used in study 1
Table S2. Demographic characteristics of the study 2 participants. Figures outside of parentheses represent raw numbers of participants.
Vignette used in study 2 (text highlighted in bold indicates those elements that differed between the ‘positive’ and ‘negative’ conditions). In the actual study materials, all text was presented in regular typeface.
Primary questions used in study 2

Supporting info item
