# Supplementary material for: Public Attitudes on the Ethics of Deceptively Planting False Memories to Motivate Healthy Behavior
Source: Appl Cogn Psychol. 2016 Sep 21;30(6):885–97. doi: 10.1002/acp.3274 (PMC5215583; doi:10.1002/acp.3274)
Supplement: Supplementary file 1 — Supporting info item [file ACP-30-885-s001.docx]

**Supplementary Materials**

Table S1. Demographic characteristics of the Study 1 participants. Figures outside of parentheses represent raw numbers of participants.

|  |  | **Country** | | |
| --- | --- | --- | --- | --- |
| **Demographic** |  | **USA** | **UK** | **Total** |
| Sex | Female | 200 (54.8%) | 215 (54.7%) | 415 (54.7%) |
|  | Male | 164 (44.9%) | 178 (45.3%) | 342 (45.1%) |
|  | Other/Prefer not to specify | 1 (0.3%) | 0 (0.0%) | 1 (0.1%) |
| Age group | 18-24 | 30 (8.2%) | 40 (10.2%) | 70 (9.2%) |
|  | 25-34 | 54 (14.8%) | 66 (16.8%) | 120 (15.8%) |
|  | 35-44 | 59 (16.2%) | 69 (17.6%) | 128 (16.9%) |
|  | 45-54 | 72 (19.7%) | 77 (19.6%) | 149 (19.7%) |
|  | 55-64 | 57 (15.6%) | 70 (17.8%) | 127 (16.8%) |
|  | 65+ | 93 (25.5%) | 71 (18.1%) | 164 (21.6%) |
| Race/ethnicity^a^ | White | 309 (84.7%) | -- | -- |
|  | Black/African American | 18 (4.9%) | -- | -- |
|  | Hispanic/Latino/Spanish origin | 23 (6.3%) | -- | -- |
|  | American Indian/Alaska Native | 1 (0.3%) | -- | -- |
|  | Asian | 10 (2.7%) | -- | -- |
|  | Some other race or origin | 4 (1.1%) | -- | -- |
|  | White | -- | 364 (92.6%) | -- |
|  | Mixed/Multiple ethnic groups | -- | 5 (1.3%) | -- |
|  | Asian/Asian British | -- | 12 (3.1%) | -- |
|  | Black/African/Caribbean/Black British | -- | 9 (2.3%) | -- |
|  | Other ethnic group | -- | 3 (0.8%) | -- |

^a^ Race/ethnicity groupings are based on the major categories used in each country’s respective national census.

**Vignette used in Study 1** (Text in italics was only presented to those participants in the “context” conditions. Text highlighted in bold indicates those elements that differed between the “positive” and “negative” conditions. In the actual study materials, all text was presented in regular typeface).

*Obesity has been linked to many of the leading causes of death: heart disease, stroke, diabetes, and some cancers. Unfortunately, obesity rates worldwide have doubled since 1980. Today, more than one in three American adults, and one in four British adults, are obese. Recent research predicts that by 2030, an additional 65 million adults in the USA and 11 million adults in the UK will be obese. This may lead to another 7.3 million additional cases of heart disease, and 669 thousand additional cases of cancer. In addition to serious health consequences, obesity is also very costly. In the USA alone, the healthcare costs of obesity were recently estimated to be around $150 billion per year. In the UK, the healthcare costs of overweight and obesity were estimated to be around £5 billion per year.*

Imagine that you are morbidly obese. Although you know that regular exercise and healthy eating habits are the best ways to improve your health, you feel that your eating habits are out of control. Desperate to improve and having tried lots of different diets without success, you seek out professional support from a therapist. The therapist informs you that there is a new therapy that improves people’s healthy eating habits and behavior. This promising therapy requires that you think and talk about your childhood memories of eating, and focus on how those memories connect to your current health situation.

Over the next few months you regularly attend therapy sessions and notice that your diet begins to improve. You now eat far more healthy foods than you do unhealthy foods. And as a result, you start to lose weight.

Now suppose that several months after your therapy ends, your therapist gets in touch with you. Your therapist explains that the highly effective therapy you received actually involved some deception. Particularly, unbeknown to you, when you talked about your childhood memories and eating habits, your therapist used suggestive interviewing methods to get you to remember **[happy/unhappy]** childhood events that never really happened to you. In particular, the therapist prompted you to remember several occasions on which you **[got really sick as a child from eating too much of unhealthy foods like ice cream and donuts / had lots of fun as a child when trying new healthy foods like asparagus and broccoli.]** You remind the therapist that you really do **remember [getting sick after eating / having fun when trying]** these **[unhealthy / healthy]** foods as a child, but your therapist explains that these are false memories, and that your family members verified they weren’t true. The therapist planted these vivid and compelling memories of **[happy/unhappy]** childhood experiences that never actually happened to you as a way to improve your eating behaviors. The therapist tells you that this technique is known as false memory therapy. **Primary** **Questions used in Study 1**

1. If I were obese, I think it would be acceptable for a therapist to deliberately plant false memories to improve *my healthy eating habits and reduce my obesity.*

Strongly disagree 1 2 3 4 5 6 7 Strongly agree

1. I think it would be acceptable for a therapist to deliberately plant false memories to improve *other obese people’s healthy eating habits and reduce their obesity. [NOTE: Q1 and Q2 presented in random order]*

Strongly disagree 1 2 3 4 5 6 7 Strongly agree

1. I think that deliberately planting false childhood memories to improve a person’s healthy eating habits and reduce their obesity is:

Completely immoral 1 2 3 4 5 6 7 Completely moral

1. I think that deliberately planting false childhood memories to improve a person’s healthy eating habits and reduce their obesity is:

Completely unethical 1 2 3 4 5 6 7 Completely ethical

1. I think that deliberately planting false childhood memories is:

Completely impossible 1 2 3 4 5 6 7 Completely possible

1. Assuming that somebody did develop false childhood memories during this therapy, I think the chance of them changing their eating habits as a result is:

Very unlikely 1 2 3 4 5 6 7 Very likely

1. In the scenario you read above, what kinds of foods did the therapist implant false childhood memories about? *[options presented in random order]*

❑ Eggs and pickles

❑ Asparagus and broccoli

❑ Ice cream and donuts

❑ Cookies and cheese

*Table S2.* Demographic characteristics of the Study 2 participants. Figures outside of parentheses represent raw numbers of participants.

|  |  | **Country** | | |
| --- | --- | --- | --- | --- |
| **Demographic** |  | **USA** | **UK** | **Total** |
| Sex | Female | 62 (51.7%) | 62 (51.7%) | 124 (51.7%) |
|  | Male | 58 (48.3%) | 58 (48.3%) | 116 (48.3%) |
| Age group | 18-24 | 16 (13.3%) | 14 (11.7%) | 30 (12.5%) |
|  | 25-34 | 22 (18.3%) | 19 (15.8%) | 41 (17.1%) |
|  | 35-44 | 20 (16.7%) | 20 (16.7%) | 40 (16.7%) |
|  | 45-54 | 22 (18.3%) | 22 (18.3%) | 44 (18.3%) |
|  | 55-64 | 19 (15.8%) | 18 (15.0%) | 37 (15.4%) |
|  | 65+ | 21 (17.5%) | 27 (22.5%) | 48 (20.0%) |
| Race/ethnicity^a^ | White | 97 (80.8%) | -- | -- |
|  | Black/African American | 4 (3.3%) | -- | -- |
|  | Hispanic/Latino/Spanish origin | 10 (8.3%) | -- | -- |
|  | American Indian/Alaska Native | 3 (2.5%) | -- | -- |
|  | Asian | 3 (2.5%) | -- | -- |
|  | Some other race or origin | 3 (2.5%) | -- | -- |
|  | White | -- | 114 (95.0%) | -- |
|  | Mixed/Multiple ethnic groups | -- | 2 (1.7%) | -- |
|  | Asian/Asian British | -- | 2 (1.7%) | -- |
|  | Black/African/Caribbean/Black British | -- | 2 (1.7%) | -- |

^a^ Race/ethnicity groupings are based on the major categories used in each country’s respective national census.

**Vignette used in Study 2** (Text highlighted in bold indicates those elements that differed between the “positive” and “negative” conditions). In the actual study materials, all text was presented in regular typeface.

Imagine that in the future, a highly effective kind of therapy exists, which changes people’s beliefs and behaviors to make them healthier. For example, it might be used to treat obesity by changing what people choose to eat, or for alcoholism by reducing how much people choose to drink. It might even be used to make people less scared of going to the dentist for a checkup.

This new fictional therapy involves therapists deliberately planting false memories in their clients’ minds – that is, creating vivid and compelling memories of events that never actually happened. Let’s call this ‘false memory therapy’. The idea is that by planting the right kind of false memories, people will be compelled to change their thoughts and behavior in ways that make them healthier.

To illustrate, picture the following scenario:

Imagine that you are morbidly obese. Although you know that regular exercise and healthy eating habits are the best ways to improve your health, you feel that your eating habits are out of control. Desperate to improve and having tried lots of different diets without success, you seek out professional support from a therapist. The therapist informs you that there is a new therapy that improves people’s healthy eating habits and behavior. This promising therapy requires that you think and talk about your childhood memories of eating, and focus on how those memories connect to your current health situation.

Over the next few months you regularly attend therapy sessions and notice that your diet begins to improve. You now eat far more healthy foods than you do unhealthy foods. And as a result, you start to lose weight.

Now suppose that several months after your therapy ends, your therapist gets in touch with you. Your therapist explains that the highly effective therapy you received actually involved some deception. Particularly, unbeknown to you, when you talked about your childhood memories and eating habits, your therapist used suggestive interviewing methods to get you to remember **[happy/unhappy]** childhood events that never really happened to you. In particular, the therapist prompted you to remember several occasions on which you **[got really sick as a child from eating too much of unhealthy foods like ice cream and donuts / had lots of fun as a child when trying new healthy foods like asparagus and broccoli.]** You remind the therapist that you really do **remember [getting sick after eating / having fun when trying]** these **[unhealthy / healthy]** foods as a child, but your therapist explains that these are false memories, and that your family members verified they weren’t true. The therapist planted these vivid and compelling memories of **[happy/unhappy]** childhood experiences that never actually happened to you as a way to improve your eating behaviors. The therapist tells you that this technique is known as false memory therapy.

**Primary** **Questions used in Study 2**

1. In the space below, please tell us whether you think it would be ethical, moral, and acceptable to deliberately plant false childhood memories to improve a person’s healthy behavior. Please explain all your reasons.
2. Your opinions about the ethics, morality, and acceptability of ‘false memory therapy’ might depend on lots of factors. For example, you might be thinking, “It would be completely unethical, unless…”, or you might be thinking, “It sounds perfectly acceptable, but not if…”.

Please tell us any factors that you might take into account when deciding whether or not ‘false memory therapy’ would be ethical, moral, and acceptable.

1. In the scenario you read, the therapist gets in touch several months after the therapy ends, to disclose that the memories ‘you’ created were actually false. Imagine that ‘false memory therapy’ would only ever work if the therapist instead lets you continue to believe that the memories are true. Would this make the therapy any more or less ethical, moral, or acceptable? Please explain all your reasons.
2. Suppose that outside of the context of therapy, somebody else—such as a parent or a teacher—tried to change a person’s healthy behaviors by using the exact same suggestive interviewing methods to plant false memories. Would this be any more or less ethical, moral, or acceptable than using these methods in the context of therapy? Please explain all your reasons.

1. I think that deliberately planting false childhood memories to improve a person’s healthy behavior is:

Completely immoral 1 2 3 4 5 6 7 Completely moral

1. I think that deliberately planting false childhood memories to improve a person’s healthy behavior is:

Completely unethical 1 2 3 4 5 6 7 Completely ethical
